# How Can We Define “Optimal Microbiota?”: A Comparative Review of Structure and Functions of Microbiota of Animals, Fish, and Plants in Agriculture

**DOI:** 10.3389/fnut.2018.00090

**Published:** 2018-10-02

**Authors:** Wakako Ikeda-Ohtsubo, Sylvia Brugman, Craig H. Warden, Johanna M. J. Rebel, Gert Folkerts, Corné M. J. Pieterse

**Affiliations:** ^1^Laboratory of Animal Products Chemistry, Graduate School of Agricultural Science, Tohoku University, Sendai, Japan; ^2^Cell Biology and Immunology Group, Wageningen University and Research, Wageningen, Netherlands; ^3^Departments of Pediatrics, Neurobiology Physiology and Behavior, University of California, Davis, Davis, CA, United States; ^4^Wageningen Livestock Research, Wageningen University and Research, Wageningen, Netherlands; ^5^Division of Pharmacology, Utrecht Institute for Pharmaceutical Sciences, Faculty of Science, Utrecht University, Utrecht, Netherlands; ^6^Plant–Microbe Interactions, Department of Biology, Science4Life, Utrecht University, Utrecht, Netherlands

**Keywords:** microbiota, agriculture, animal husbandry, aquaculture, rhizosphere, phyllosphere, agricultural immunology

## Abstract

All multicellular organisms benefit from their own microbiota, which play important roles in maintaining the host nutritional health and immunity. Recently, the number of studies on the microbiota of animals, fish, and plants of economic importance is rapidly expanding and there are increasing expectations that productivity and sustainability in agricultural management can be improved by microbiota manipulation. However, optimizing microbiota is still a challenging task because of the lack of knowledge on the dominant microorganisms or significant variations between microbiota, reflecting sampling biases, different agricultural management as well as breeding backgrounds. To offer a more generalized view on microbiota in agriculture, which can be used for defining criteria of “optimal microbiota” as the goal of manipulation, we summarize here current knowledge on microbiota on animals, fish, and plants with emphasis on bacterial community structure and metabolic functions, and how microbiota can be affected by domestication, conventional agricultural practices, and use of antimicrobial agents. Finally, we discuss future tasks for defining “optimal microbiota,” which can improve host growth, nutrition, and immunity and reduce the use of antimicrobial agents in agriculture.

## Introduction

Today, biologists in agricultural science, regardless of the organism of their interest, focus significant attention on the microbiota, i.e., the complex communities of microorganisms colonizing host animals, fish, and plants (1). Meta-analyses of 16S rRNA genes from different body parts of animals, fish, and plants are frequently performed expecting that some changes of microbiota will explain the effectiveness of treatments such as feed changes, fertilizer amendment, or gene knockouts on host organisms, which have been conducted with aims to improve productivity and sustainability in agriculture (2). However, it is often the case that no apparent changes are observed in the microbial structure corresponding to the specific treatment, or if present, the functions of the responding microorganisms are not well-known [e.g., (3–5)]. Besides, it is often difficult for researchers in agricultural sciences to exploit the microbial data to improve the host factors because of the lack of definition and criteria of “optimal microbiota” in animals, fish, and plants.

Compared to a large body of studies on microbiota of human subjects (6) or experimental models using rodents(7), zebrafish (8), or *Arabidopsis* (9), there are a very limited number of studies on economically important animals, fish, and plants. Microbiota datasets obtained from livestock animals, aquaculture fish, and crop plants grown may significantly be affected by complex environmental factors such as climates, cultivation scales, and uses of antibiotics and fertilizers, which can vary between different countries and regions. Besides, the microbiota of agricultural organisms may also reflect the great variability of host species and genotypes, biological functions at different developmental stages, and macro- and microstructures of the colonizing sites, which are not thoroughly studied as the laboratory models. Due to the overall limited understanding of the microbiota in agricultural ecosystems at this point, it is not an easy task to define “optimal microbiota,” which can optimize the growth, host nutrition, and immunity of agricultural organisms.

The importance of understanding the structure and functions of microbiota in agriculture is also widely discussed in the context of the spread of antimicrobial resistance (AMR) from agricultural sites to human society (10). While manipulation of microbiota is a promising strategy to tackle the AMR (11), it is prerequisite for researchers to interpret and exploit the rapidly expanding datasets of the microbiota in animals, fish, and plants in agriculture with a more generalized view. By sharing knowledge on the ecophysiology of microbiota in different host organisms with respect to their structure and metabolites and understanding how the host factors and ambient conditions can alter them, we would be able to refine targets of microbial manipulation and reduce uses of chemicals and antimicrobial agents in agricultural fields.

The aim of this review is to summarize and generalize the current knowledge on the microbiota on animals, fish, and plants in agriculture with emphasis on structure and functions of bacterial communities, which may contribute to the health of the host organisms and can strongly be impacted by agricultural practices such as uses of antimicrobial agents. We finally provide important yet overlooked aspects of microbiota in animals, fish, and plants in agriculture, which should be considered in future studies to reach the goal of defining the “optimal microbiota.”

## Structure and function of microbiota of animals, fish, and plants

### General overview of microbiota of animals, fish, and plants

**Figure 1:**
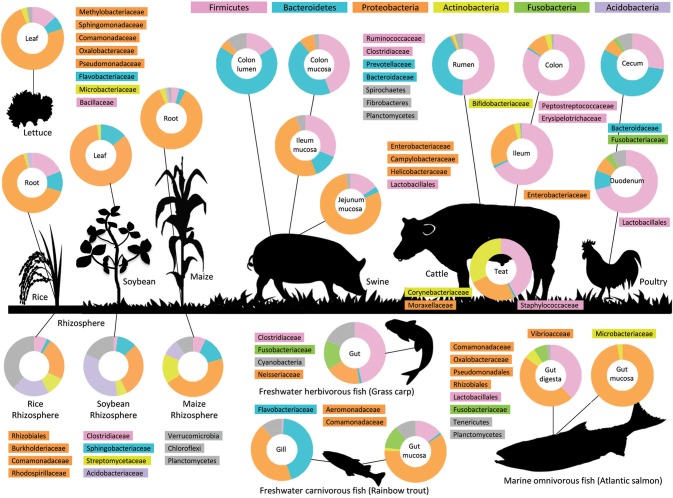
Microbiota in agriculture. The figure provides an overview of the bacterial composition of the microbiota of different parts of livestock animals, gill and intestines of fish, and phyllosphere and rhizosphere of plants at the phylum-level (pie-charts) and lower taxonomic levels. The data sources are 16S rRNA or metagenomic analyses of intestinal samples from pigs (14, 15), cattle (3, 16), chicken (17), Atlantic salmon (18), grass carp (19), gill and mucosal samples from rainbow trout (20), leaf samples from lettuce (21), leaf and rhizosphere samples from soybean (22, 23), root and rhizosphere samples from maize (24), rice (25, 26).

The body of organisms provides a wide variety of ecological niche, in which the environmental conditions such as temperature, pH, and oxygen level as well as nutrition availability affect the composition of microbiota residing there. While archaea and eukaryotic microorganisms such as fungi and protozoa account for a significant proportion of microbiota in the plant rhizosphere (12) and cow rumen (13), bacterial communities have been primarily focused on in many studies in agricultural science in terms of their functional contribution to host nutrition and health. The 16S rRNA gene-based approach with a next-generation sequencing platform has revealed diversity and dynamics of bacterial communities colonizing animals, fish, and plants in agriculture, which have enabled us to grasp a general overview of compositional similarities and differences of microbiota among these organisms (Figure 1). Microbiota of animal, fish, and plants are highly diverse and can harbor up to 20 bacterial phyla, but it is a common trait that three phyla: Proteobacteria, Firmicutes, and Bacteroidetes, dominate the bacterial community (Figure 1). Less abundant phyla include Actinobacteria, which are commonly found but variable at lower taxonomic levels (e.g., Streptomycetaceae, Microbacteriaceae, and Corynebacteriaceae), while Fusobacteria and Acidobacteria are more specified to animal/fish and plants, respectively (Figure 1). Fusobacteria can represent a major bacterial group of “core gut microbiome” of some marine and freshwater fish (8, 27). Chloroflexi, Cyanobacteria, Planctomycetes, Spirochaetes, and Verrucomicrobia sporadically occur as subdominant phyla (Figure 1).

The high abundance of Proteobacteria in animals, fish, and plants (Figure 1) reflects the advantages of facultative anaerobes in the host proximity, where strict anaerobes are exposed to the risk of oxygen toxicity but strict aerobes may face a severe competition over oxygen as an electron acceptor. Such oxic-anoxic interface is ubiquitous as microenvironments in and around the host organism and is an important determinant of the composition of microbiota (28). Facultative anaerobic bacteria have highly flexible metabolic properties; they are able to generate energy by fermentation or use inorganic nitrogen compounds such as nitrate as an alternative electron acceptor when oxygen is depleted from the environment. Under oxic conditions, they grow rapidly using oxygen and break down and build up a wide variety of organic compounds, which essentially change the surrounding organochemical conditions (29). Such exceptional adaptability to multiple environmental conditions, which have been characterized by their high genetic and phenotypic plasticity, enable Proteobacteria to be specialists of host association, as represented by major target symbionts and pathogens in agriculture (30–32).

While Proteobacteria are ubiquitous and their association is often described to be opportunistic, they show apparent host specificities in some microbiota. In fish intestinal microbiota, Aeromonadaceae (Gammaproteobacteria) represents the most abundant symbionts in freshwater fish, while Vibrionaceae (Gammaproteobacteria) replace the occupation in marine fish (27, 33). In microbiota of livestock animals, the proteobacterial community is predominated by Enterobacteriaceae, followed by Campylobacteriaceae and Helicobacteraceae, which are a major source of foodborne diseases of human (3, 34, 35).

Plant phyllosphere has been found to be dominated by strict aerobes, represented by Methylobacteriaceae and Sphingomonadaceae (Alphaproteobacteria), but their abundance is low in the plant root, being replaced by a large diversity of facultative anaerobes (36). Interestingly, microbiota of plants and fish have several groups of facultative anaerobes in common. Multiple apertures of fish, i.e., skin, gills, and gut, are constantly in contact with ambient water, each of which is covered with thick mucus biofilm, which bears resemblance to the plant root system. Comamonadaceae and Oxalobacteraceae (Betaproteobacteria) as well as Flavobacteriaceae (Bacteroidetes), which dominate the leaf-to-root microbiota of plants, are also abundant in fish mucus, especially in gill microbiota [Figure 1; (36, 37)]. Also, bacteria known as plant growth-promoting microbes (PGPM) such as Pseudomonadaceae (Gammaproteobacteria) and Rhizobiales (Alphaproteobacteria) are also frequent colonizers in fish intestinal microbiota (33, 38). In contrast, these bacterial groups are not commonly found in animal intestinal microbiota.

Firmicutes population in animals, fish, and plants can be roughly classified into two groups: Lactic acid bacteria from the orders Bacillales and Lactobacillales, and anaerobic fermentative bacteria affiliated with Clostridiales such as Clostridiaceae and Ruminococcaceae. The former represents microbiota in oxic- to microoxic regions like plant phyllosphere, fish mucosa, and small intestines of animals, while the latter represents anoxic fermentative digestive tracts like rumen and large intestine (Figure 1). Lactobacillales is one of the most frequently found and most studied bacterial groups in animal and fish microbiota with some family-level variations, as Lactobacillaceae is a stable and important colonizer in the small intestine of pigs (39, 40) and chickens (17), Carnobacteriaceae [rainbow trout; (20)], and Leuconostocaceae [Atlantic salmon; (18)] are characteristic in fish microbiota, and Enterococcaceae and Streptococcaceae are generally found both in animals and fish microbiota (41). Clostridiaceae is phylogenetically and functionally diverse and widely distributed in anaerobic environments from plant rhizosphere to animal and fish intestines (42). Studies on *Clostridium* spp. from various origins have shown their metabolic versatility and dexterous switching of their fermentation pathway in response to environmental changes (43, 44).

Bacteroidetes colonizing animals, fish, and plants can also be affiliated with two types: aerobic Flavobacteriaceae, which is adapted to the oxic interface of plants and fish as mentioned above, and anaerobic fermentative bacteria such as Bacteroidaceae and Prevotellaceae (Figure 1). Flavobacteriaceae is often recognized as threatening pathogens of animals and fish (45), but it also represents PGPM and has been introduced to industrial applications (46). Bacteroidaceae and Prevotellaceae are important primary fermenters in animal and fish intestinal tracts, through which complex carbohydrates derived from plants and undigested proteins enter the microbial metabolic network and provide soluble sugars and amino acids available for all type of cells (47). They dominate the rumen and colonal microbiota of animals (48, 49) but are rarely found in fish and plants, which suggests their speciation to animal digestive tracts.

### Relationship between intestinal microbiota composition and host nutrition of animals and fish

Microbiota of animals and fish associated with different host physiological conditions have been widely studied to elucidate the relationship between the structure and functions of microbiota and host nutritional health. Although results are variable across studies, which may be attributed to different experimental designs, analytical methods, or individual variations, a few general aspects can be inferred from recent studies.

In animal husbandry, feed efficiency and growth performance are often focused on as the most important physiological factors. Recent studies on pig microbiota have reported the enrichment of Clostridiales, as well as microbial functional genes involved in fermenting dietary polysaccharides and amino acid metabolism, are positively associated with porcine feed efficiency (50, 51). Similarly, positive correlations of Clostridiales (family Lachnospiraceae) to good feed efficiency have also been found in the cattle rumen (49) and chicken caeca (52). In fish, the intestinal microbiota of prebiotics-treated fish with improved growth performance also showed an increased number of *Clostridium* spp. (53). All of these studies have attributed the positive effects of Clostridiales on the host feed efficiency and growth performance to the high energy yields by the production of short-chain fatty acids (SCFA) such as butyrate, which has also been suggested in human gut microbiota studies (54).

Lactobacillales are thought to improve feed efficiency of animals and multiple *Lactobacillus* strains are widely used as feed additives especially in pig farming (55). While studies have suggested a positive correlation of *Lactobacillus* to a better feed efficiency of cattle, chickens, and fish (17, 49, 53), contrasting effects of *Lactobacillus* spp. on growth performance have been also reported on chicken (17, 52, 56). Therefore, species- and strain-level variation should be considered when the abundance of *Lactobacillus* strains is used as a criterion for evaluating the health and growth performance of animals and fish.

Both in animals and fish, intestinal microbial colonization has been shown to promote epithelial cell turnover and regulate transcription of genes involved in nutrient metabolism and immunity, and the corresponding gene modules are universally conserved between mammals and fish (57–59).

### Rhizosphere vs. phyllosphere: difference of microbiota composition

Plant microbiota significantly differed from those of animals and fish, in that in addition to the complex bacterial community, a large variety of archaea as well as eukaryotic macro- and microorganisms can directly and constantly affect the health of the host plant (12, 60). In response to this challenge, plants have a finely regulated immunological capacity, which recognizes different exogenous molecules and responds by activating specific defense mechanisms (61). The plant rhizosphere is home of a high density [10^10^−10^12^ cells per gram soil; (62)] of microorganisms and a large pool of microbial metabolites influence the nutritional conditions of the host plant as well as the composition of microbial populations (63). In contrast, microbiota in the plant phyllosphere, i.e., leaf and root, are enriched with restricted groups of bacteria. As mentioned already, Proteobacteria and Flavobacteria have been found as endophytes or epiphytes of the host plant, while any other bacterial phyla dominating the rhizosphere, such as the phylum Acidobacteria and Firmicutes, or prominent rhizobacteria such as Streptomycetaceae (Actinobacteria) and Burkholderiaceae (Betaproteobacteria) are segregated at different levels of the phyllosphere (64). This suggests the existence of a “selecting gate” between the rhizosphere and the phyllosphere, or different compartments of the plant root (65).

Studies across different plant species including the *Arabidopsis* model indicate that the enrichment of Proteobacteria is a common trait in the plant phyllosphere (Figure 1). However, the enriched bacterial species, i.e., members selected by the host plant, seem to differ significantly between plant species. Pseudomonadaceae (Gammaproteobacteria) and Streptomycetaceae (Actinobacteria), which are frequent colonizers in the root of *Arabidopsis*, are not found in grass plants such as barley, maize, and wheat (24, 66). Also, leaf microbiota between plant species can be very different, as exemplified that Enterobacteriaceae, Bacillaceae, and *Pantoea* spp. dominating spinach and lettuce leaves are not abundant in *Arabidopsis* (21, 67). While the bacterial composition of microbiota of the phyllosphere of economically important plants is very limited compared to that of the rhizosphere (36, 68), the composition of microbiota in different plant compartment may provide useful insights into site-specific selection mechanisms of the host plant.

### Acquisition of microbiota in the early life of animals, fish, and plants

In animals, the intestinal immunity is known to be developed in the course of frequent interaction with microbiota, which are formed and fluctuated in response to the host dynamics (69). It has been shown that the early-life transfer of microbiota from the mother to the child via the birth channel and colostrum milk can impact on subsequent intestinal microbial diversity and immune processes in piglets (70). The transition from nursing, weaning to conventional diets can dramatically affect intestinal microbiota. Milk provides immunological factors such as Immunoglobulin A (IgA), leukocytes, and peptides, which suppress inflammatory cytokine expression, and lactose and oligosaccharides contained in milk can stimulate the growth of early-colonizing microorganisms such as lactic acid bacteria. (71). Comparative analyses of the intestinal microbiota of nursing and weaning piglets have shown that the dietary change from sow milk to a starter diet composed of plant and animal-based components has a significant impact on the microbial structure as well as its functional capacities (34). In their study, Enterobacteriaceae, Bacteroidaceae, and Clostridiaceae dominating the nursing piglets almost disappeared as the piglet diet shifted to a starter diet, which has been characterized by the dominance of Prevotellaceae and Ruminococcaceae associated with plant polysaccharide degradation. The early colonization and subsequent disappearance of Enterobacteriaceae as well as the maturation of microbiota associated with the domination of plant-polysaccharide degraders in the early life have also been commonly found in other mammals including human (72) and also in chickens (73). Proliferation of pathogenic members of Enterobacteriaceae can be regulated by selective binding activities of host-derived IgA, which seems to be one of the most important mechanisms affecting early development of intestinal microbiota in animals (74).

Fish develop from eggs that are directly exposed to microorganisms in their surroundings. The eggs are quickly coated with microorganisms present in the surrounding water, of which some have been shown to protect the eggs from infection with oomycete *Saprolegnia*, a deleterious pathogen causing economic loss in the salmon industry (75). Since fish represents the largest number of vertebrates (>28,000 species), a lot of interspecies variation may exist based on the receptors or binding moieties on the egg surface. Although microbiota of fish larvae is poorly understood (76, 77), some studies have suggested that the microbiota composition of fish larva greatly depend on the microorganisms present on the eggs, in the live feed and rearing water (78, 79). Since microorganisms are able to enter the fish larvae before it starts feeding (3–4 days after fertilization), initial microbial infection in the larval intestine probably occurs before the feed specific species grow to abundance [(76, 80) Lopez Nadal, unpublished observations)]. In early life stages of Coho salmon, *Pseudomonas* sp. present on the eggs has been predominantly found in the juvenile gastrointestinal tract, but not in the culture water or food, which suggests that a maternal transfer may occur in the early developmental stages of the salmon (81).

The development of seedlings from largely sterile plant seeds is one of the most critical stages of a plant's life cycle. Yet, very little is known about the role of microbiota in the early life of plants (82). Starting inoculum on the ripening seed may be important for the establishment of microbiota and preliminary enrichment of the soil microbiota by the parental plant will form ideal environments for germination of seeds in the same soil. Interestingly, it has recently been shown that diseased plants can recruit themselves a consortium of beneficial, immune-stimulatory microbes from the soil environment and let them colonize germinating seedlings, which suggests that plants are capable of selecting soil microbiota for protecting a successive generation of plants against the causal agent of the disease (83, 84).

### Post-translational host modulation by microbiota

Host epigenomics has recently been shown to be one of the most important factors significantly affected by microbiota. Anti-inflammatory effects of some intestinal microorganisms such as *Clostridium* spp. have been attributed to their metabolite butyrate, an epigenetic substance known to inhibit activities of histone deacetylases and modulate gene expression patterns of host animals (85). Also in fish, promotion of resistance to viral infection of conventionally reared zebrafish has been shown to be associated with microbe-induced epigenetic changes in the host (86). In plants, not only bacterial pathogens but also fungal and other eukaryotic organisms have been known to manipulate their host epigenetically to favor themselves (87). While low-molecular-weight microbial metabolites such as SCFAs and polyamines from mammalian intestinal microbiota have been shown to be involved in various epigenomic mechanisms in the mammalian host (88), modulatory effects of microbial structural components such as LPS, peptidoglycan, and exopolysaccharides from microbiota in most agricultural organisms have not yet been well-studied except for those from some pathogens and probiotics (see below). Molecular mechanisms how microbiota modulates host epigenomics have recently been attracting major attention, which may also contribute to understanding functions of microbiota in animals, fish, and plants in agriculture (89).

## Microbial metabolites: beneficial and deleterious effects of metabolites produced by microbiota of animals, fish, and plants

“Optimal microbiota” of agricultural organisms are expected to provide beneficial effects on their host nutritional health and immunological resistance. Microorganisms influence the host health by producing a large variety of metabolites, which can have both beneficial effects and detrimental effects on the host physiology (Table 1).

**Table 1:** Important microbial metabolites and their effects on host animals, fish, and plants.

**Microbial metabolites**	**Examples**	**Hosts^a^**	**Beneficial effects**	**Detrimental effects**
Short-chain fatty acids (SCFAs)	Butyrate	A, F	Energy homeostasis anti-inflammatory effect, improve intestinal barrier	Mucosal disruption
	Propionate			Neurotoxicity
Organic acids	Lactate	A, F,	Increase butyrate production	Acidosis, inflammation, neurotoxicity,
	Succinate	A, F, P	Glycemic control, feed PGPM, mineral solubilization	Feed pathogens
Ammonia and amino acid derivatives	Ammonia, ammonium	A, F, P	Nitrogen nutritional source, pH neutralization	Inflammation, mucosal damages, increase oxidative stress
	Biogenic amines	A, F	Synthesis of neurotransmitter (serotonin)	Production of uremic toxins, carcinogenesis
Signaling molecules acting on the host	IAA, 2,4-DAPG, GABA	A, F, P	Growth promotion, anti-inflammation	
Signaling molecules acting on other microbes	AHL, AI-2		Maintenance of microbial structure (e.g., biofilm formation), cell-to-cell communication between microbes	
Antimicrobial compounds	Bacteriocins, RiPPs	A, F, P	Defense against pathogens, immunomodulatory effects	Cytotoxicity
Vitamins	Vitamin B_12_, vitamin K, D	A, F	Provisioning of host nutrition, immunomodulation	
Microbial cellular components	LPS, Polysaccharide A	A, F, P	Immunomodulation, maintenance of intestinal homeostasis	Inflammation

a *A, animals; F, fish; P, plants*.

### Short chain fatty acid (SCFA)

Fermentative microorganisms break down carbohydrates and proteins into SCFA. In the gut of animals and fish, major SCFAs produced by fermentative microorganisms are acetate, propionate, and butyrate, while relatively low amounts of formate, valerate, caproate, and branched-chain SCFAs, i.e., isobutyrate, 2-methyl-butyrate, and isovalerate, which are used as a marker of undesired intestinal protein fermentation (90), are also present (91, 92). SCFAs can modulate the gene expression of the host epithelial cells in multiple ways and their physiological concentrations may significantly affect the host nutritional health and immunity (93, 94). SCFAs produced by gut microbiota are known to serve as a major energy source for ruminant animals, which consume cellulose fibers and complex carbohydrates as the main diet, but also play a crucial role for young monogastric animals for maintaining the body weight after weaning (95, 96). Additional roles of SCFA include defense mechanisms, mineral solubilization, and the anti-inflammatory effects (97, 98). SCFA produced by intestinal microbiota improve intestinal barrier functions and suppress inflammation through signaling pathways such as activating G-protein coupled receptors, inhibiting histone deacetylase, stimulation of histone acetyltransferase activity, and stabilizing hypoxia-inducible factor (HIF), which have been extensively studied with rodent models (99, 100).

While SCFAs produced by intestinal microbiota are generally considered to be beneficial to the host, excessive SCFAs can cause intestinal injuries in animals with premature or weakening mucosal conditions (101, 102). Formate, which concentration increases along with dysbiosis, can enhance the growth of unwanted enterobacterial pathogens (103). High levels of propionate are often found in human and animals with psychological and behavioral disorders and thought to have a neurotoxic potential (104, 105).

### Lactate and other organic acids

Lactate is an important intermediate in anaerobic fermentation of carbohydrates. While host-derived lactate has been known for regulatory functions on the energy homeostasis and brain metabolism (106, 107), lactate produced by microbiota may also play important roles in the intestinal ecosystem, such as turnover of host epithelial cells (108), in addition to their role as a major food source for other SCFA producing bacteria (109). In the small intestine of animals and fish, lactic acid bacteria such as Lactobacillales (Figure 1) are known to produce lactate as a primary metabolite, while *Turicibacter* (Erysipelotrichaceae) represent the major lactate producers in the large intestine. Residuous oxygen may increase relative abundance of intestinal lactic acid bacteria, which generally show high tolerance against oxygen (110, 111), and lactate productions and consumption profiles may differ significantly between upper and lower intestines (112).

Succinate is another major organic acid released from microbiota during carbohydrate fermentation. Prevotellaceae and Veillonellaceae, which are predominant bacterial groups in the rumen and in the colon of pigs, are major succinate producers. A large variety of bacteria including Enterobacteriaceae and Clostridiaceae can grow on succinate, and succinate accumulation would increase a risk of infection by pathogenic bacteria (113). Recent studies have reported succinate production by gut microbiota is strongly correlated to the metabolic fluctuation of host animals (114, 115).

Accumulation of lactate and succinate has been reported in the intestine of pigs with gastric problems (116, 117), which has been shown to be inversely related to the SCFA concentrations (118). Increased concentrations of lactate and succinate can cause a decline in pH and drastic changes in metabolic patterns in animal and fish intestines, which can lead to deleterious outcomes such as acidosis and inflammation (119). To avoid this, a rapid turnover of lactate by gut microbiota seems to be crucial for intestinal homeostasis in animals and fish (28).

Plant root exudates contain a high amount of organic acids, such as citrate, succinate, and malate, which can significantly affect the composition of the microbial community in the rhizosphere (120, 121). The high amount of organic acids exudated from the host plant feed and control proximal microbiota consisting of plant growth-promoting microorganisms (PGPM) as well as pathogens, and the microbiota in rhizosphere may also affect the concentration of organic acids excreted from the plant host by modulating their regulatory genes (122, 123). As organic acids can affect the growth and plant-promoting activities of PGPM, e.g., suppressing phosphate stabilization (124), the concentration of organic acids should be well fine-tuned by the host-microbe regulatory network (125).

### Ammonia and amino acid derivatives

Ammonia (NH_3_) and ammonium (${\text{NH}}_{4}^{+}$) play an important physiological role in the body of animals, fish, and plants as it provides usable forms of nitrogen required for the synthesis of DNA, RNA, and proteins. Ammonia not only serves as a major nitrogen source but also are responsible for buffering the ecosystem such as rumen by neutralizing excess acids. Many bacteria are able to generate ammonia via protein or peptide degradation and N_2_ fixation. Fixed atmospheric N_2_ in the ${\text{NH}}_{4}^{+}$ form is an important source of nitrogen in the soil ecosystem, which concentration in agricultural soils is approximately between 20 and 200 μM (126), and many plants are highly dependent on endophytic or rhizospheric nitrogen-fixing bacteria for their nitrogen demands. Ammonia is also an important metabolite in the microbiota of animals and fish and millimolar level concentration of ammonia can be generally found in the intestinal ecosystem [e.g., 10–70 mM in colonic lumen; (127)]. Many bacteria such as *E. coli* and *Bacteroides* spp. are known to require ammonia or ammonium for their growth in the intestinal system, while they are able to provide amino acids and their derivatives to other intestinal bacteria and the host (29, 128).

Toxicity of ammonia (NH_3_) and ammonium (${\text{NH}}_{4}^{+}$) from microbiota poses a risk to the host as well. When excess protein is present in the intestine, ammonia production by microbial deamination will exceed microbial ammonia assimilation (129). Urea produced by the host animals is also converted to ammonia and further to ammonium hydroxide by microbiota, which can elevate luminal pH at the level of causing mucosal damage and irritation (130). Accumulated ammonia has multiple adverse effects on host epithelial cells (129). Ammonium toxicity is also documented in plants, but the cause for this phenomenon and involvement of microbiota is still unknown (131).

Increased protein and peptide concentrations in a microbial ecosystem may facilitate active amino acid conversion to various nitrogenous derivatives. Many facultative and obligate anaerobic bacteria ferment amino acids into a wide variety of intermediate metabolites such as indoles, phenols, cresols, and their derivatives as well as biogenic amines (132). Biogenic amines such as tyramine, putrescine, histamine, methylamine, and tryptamine, are produced by decarboxylation of amino acids, which have significant physiological and toxicological functions in eukaryotic cells (132, 133). Biogenic amines serve as precursors of various bioactive compounds, which can directly regulate physiology and behavior of the host. For example, tryptamine, a β-arylamine neurotransmitter derived from tryptophan metabolism, influences modes of serotonin production in enterochromaffin (EC) cells and therefore affect host behavior (134, 135).

### Secondary metabolites

Secondary metabolites from microbiota such as tryptamine, which can serve as hormones or signaling molecules (136) to “control” the host physiology and behavior, are also known for plants. Indole-3-acetic acid (IAA), one of the most important plant growth regulators, is also derived from the tryptophan metabolism of PGPM such as Pseudomonadaceae (137). Pseudomonadaceae are also known to produce a wide variety of secondary metabolites including antibiotic compounds and siderophores, which can protect the host plant from invasive pathogens not only in the rhizosphere but also in phyllosphere (138, 139). Specific secondary metabolites of Pseudomonadaceae such as 2,4-diacetylphloroglucinol (2,4-DAPG) are of special interests for controlling specific plant–microbe interaction (140). In animals and fish, secondary metabolites produced by gut microbiota such as gamma-aminobutyric acid (GABA) are likely to have more general but significant influence on physiological and psychological properties of the host (141).

Antimicrobial compounds such as bacteriocins, siderophores, and lipidopeptide biosurfactants enable some microorganisms to outcompete and eliminate pathogens and shape the structure of microbiota by also affecting the host immunity (139, 142, 143). Although bacteriocins and siderophores have been well-documented in some beneficial or pathogenic strains, genes encoding these compounds could be commonly found in a wide range of microorganisms (144). In human microbiota, ribosomally synthesized post-translationally modified peptides (RiPPs), which include lantibiotics, thiazole/oxazole-modified microcins (TOMMs) as well as thiopeptides antibiotics, are one of the most widely distributed and variable microbial metabolites (145).

Signaling molecules known as autoinducers play important roles in cell-to-cell communication between microorganisms and shape the synchronized behavior of microbial community such as biofilm formation (146). In contrast to the well-known quorum-sensing molecule AHL (N-acyl homoserine lactone), which are produced as virulence factors by many gram-negative pathogenic bacteria and probably uncommon in healthy intestinal microbiota in animals and fish (147, 148), AI-2 (autoinducer 2) are present in many intestinal bacteria such as Firmicutes and Bacteroidetes and known to modify the structure and behavior of intestinal microbiota (149, 150).

### Vitamins

Animals depend on their gut microbiota for various vitamins, which are often deficient in their normal diet. Deficiencies in vitamin B_12_ and other B-complex vitamins, as well as vitamin K and D in animals and fish has been correlated to the absence of intestinal microorganisms producing those vitamins (151, 152). In addition to the crucial role for the host nutritional health (153–155), vitamins formed by microbiota are also provisioned to other microorganisms in proximity thereby supporting the cross-feeding metabolic network in gut microbiota (152). Some vitamins are also known to participate in host epigenomic mechanisms by altering the transcriptional machinery of the host cells (88). While most plants can synthesize vitamins and do not depend on their microbiota for their vitamin requirements, some algae have been known to benefit from the microbial provision of vitamin B_12_ (156).

### Microbial cellular components

Microbial metabolites affecting host health also include structural compounds of microorganisms themselves. Exo- and lipopolysaccharides (LPS), peptidoglycan, flagellin, and some unique peptides and nucleic acids released from the microbial community, which are often collectively called as microorganism-associated molecular patterns (MAMPs), are specifically detected as “non-self” and distinguished by pattern recognition receptors (PRRs) of the host cells and trigger immune responses in animals, fish (86), and plants (157, 158). It has long been recognized that MAMPs from pathogens play a crucial role for host immunity in animals and plant (96, 159–161), but recent studies have revealed that MAMPs from commensal microbiota may also control the host immune system to maintain intestinal homeostasis (162). Common intestinal residential bacteria such as *Clostridium* and *Bacteroides* have been shown to stimulate the production of cytokines such as IL-6 and TNFα that protect intestinal tissues from injury (163), and also to induce the proliferation of immune cells such as FOXP3^+^ regulatory T (Treg) cells (164). Although MAMPs required for induction of each host factor are not well-understood, species-specific polysaccharides such as Polysaccharide A found on the capsule of *Bacteroides fragilis* may play important roles for initial binding and recognition to the host cells (165).

In the model plant *Arabidopsis thaliana* it was recently shown that MAMPs from beneficial root microbiota members are similarly recognized by the plant immune system as MAMPs from pathogens, but the downstream immune response was suppressed by so far unknown mechanisms (89).

## Influence of agricultural management practices on microbiota in animals, fish, and plants

Recent comparative studies on gut microbiota between urban and hunter-gatherer human population have suggested continuous decreases in microbial diversity over generations during worldwide industrialization (166–168). Similar changes, i.e., loss of diversity in domestic vs. wild counterparts, have been documented in primates and Przewalski's horses (169, 170), but the diversity level of gut microbiota has been found to be consistent in mice (171) and a vice versa situation has also been observed in cloacal microbiota in parrots (172). Nevertheless, many studies have shown that the reduced diversity of gut microbiota is characteristic to many diseases and disorders in human [e.g., (173, 174)], therefore the loss of diversity in gut microbiota over generations may have negatively affected the health of not only human but also other animals and fish. Although reduced microbial diversity is not often discussed for plants, the long-term agricultural practices may have served as strong selective pressures on the microbiota of the phyllosphere and rhizosphere (175, 176). Supplementation of the “lost” population could improve host fitness, as has been shown in mice (177), but the cause of the loss of certain microbial groups and its consequences are not fully understood. For optimizing microbiota in agricultural organisms, it is important to evaluate how domestication and agricultural management practices can affect the microbiota and host nutritional health and immunity.

### Domestication

While some livestock animals and farmed fish have evolved into domestic species distinct from wild relatives, studies on the microbiota of wild representatives of agricultural organisms provide insights into how domestication may have affected the microbial composition of agricultural organisms. For example, a comparative study of domestic pig microbiota with that of wild boars has revealed that *Lactobacillus* spp. and Enterobacteriaceae, which are considered to be dominant bacterial groups in pig intestinal microbiota (178), are not common in wild boars (179). Interestingly, recently domesticated wild boars have been found to harbor Enterobacteriaceae as a major group, which collectively suggests that gut microbiota of domestic pigs may reflect the recent agricultural management practices (179). Since the increased abundance of Enterobacteriaceae has been reported to be correlated to post-weaning diarrhea (180), agricultural management is likely to have a significant impact on the health of domestic pigs via the fluctuation of gut microbiota. In cattle, inoculation with bison rumen contents has been shown to increase protein digestibility and nitrogen retention but not fiber digestibility, which suggests that microbiota of ancestors of livestock animals may have had higher capacities to extract nitrogen nutrition from crude materials (181).

While a study on gut microbiota of laboratory-reared and recently-caught zebrafish has shown little influence from domestication on intestinal microbiota of fish and shrimps (8, 182), some bacterial groups in wild fish have been found to disappear upon captivity (183) and therefore careful investigation should be needed in future studies.

Plant microbiota can also be affected by domestication, i.e., plant breeding in combination with yield-increasing agricultural practices and the use of chemical fertilizers and pesticides, which has resulted in the selection of specific plant traits maximizing profitable functions from the root microbiome (184). Studies have shown distinct features of the microbial community associated with wild and domesticated crop species such as rhizosphere microbiota from sugar beets (185) and endophytic population from grapevines (186). Nevertheless, plant hosts respond to various microbial factors by changing their physiology and thereby can modulate their microbial composition (187), it is important to obtain more insights into the physiological and structural differences between wild and domesticated plant species.

### Agricultural management practices

Agricultural management practices include multiple and long-term stress factors such as selective breeding, confinement, nutritional changes, close contact with people, and antimicrobial usage, all of which can affect the composition of microbiota to a greater extent.

Selective breeding produces a new type of organism with a phenotype different from its parental organisms, which can affect the composition of the host-specific microbiota. Gut microbiota in livestock animals including cattle and pigs have been reported to show a host-specificity and habitability over generations (188, 189), which suggests that the host genetics are correlated to microbial structure and functions (190). Although fish gut microbiota are largely affected by ecological factors, several studies have shown host selection plays an important role in shaping intestinal and gill microbiota (191, 192). In plants, rhizosphere microbiota has been shown to have specific profiles unique to its host plant species, genotype, and cultivar (193, 194).

Confinement such as indoor breeding and aquaculture has been reported to affect microbiota of animals and fish to various extents. There is no clear evidence on how housing systems (indoor vs. outdoor) can affect microbiota of animals, since previously reported changes in gut microbiota in response to different housing methods can be better explained by dietary changes (195, 196). As intestinal tracts of fish are constantly exposed to water and a large number of microorganisms in their surroundings, it is not surprising that the conditions of aquaculture such as water quality (e.g., salinity) and external microbial community significantly affect gut microbiota of fish (197–199).

Nutritional changes including grazing, feeding, and weaning in animals or fertilizer amendment for plants, are one of the most important factors shaping the structure and functions of microbiota in agriculture. Availability of microbiota-accessible organic compounds is a crucial determinant for the survivability of individual microorganisms in the host systems (200). In livestock animals, starch grains, plant fibers and crude proteins in feed are digested by rumen or colonic microbiota to different degrees, which can essentially change the structure and functions of gut microbiota and the host nutritional health as already mentioned above (51, 201, 202). Weaning can cause serious fluctuation of rumen and intestinal microbiota of young animals (34, 203), which can occasionally lead to dysbiosis and post-weaning diarrhea (204). Also similar to animals, dietary changes such as feeding high-cellulose diet has been shown to increase cellulolytic bacteria in the fish intestine (205, 206), and relationships between the dietary components and gut microbiota are extensively studied (207). Continuous cropping and fertilizer amendment can modulate nutritional status in the agricultural soil and affect plant microbiota (208). The growth inhibition caused by the continuous cropping, such as the imbalance of inorganic nutrients and prevalence of pathogenic fungi, could be mitigated by native microbiota (209), which suggests the resilience of agricultural soils highly depend on their microbial activities.

Continuous close contact with human and animals seems to allow inter-species transmission of certain bacterial groups even between the intestinal microbiota. As the microbial composition in human gut has been found to be affected by adjacent livestock animals or companion animals (210, 211), the microbiota of animals, fish, and plants could be affected by the human microbial assemblage. The predominance of *Bifidobacterium* spp. in the modern human gut microbiota reflecting the dietary transition from fiber-rich plant-based diet to western diet (212), has also been observed in animals, which have experienced close contact with humans (213).

### Influence of usage of antimicrobial agents on microbiota in agriculture

Antimicrobial agents have been used as a common agricultural practice over the decades, which aims not only to treat infectious diseases of animals and fish but also to promote growth and improve productivity (214). The worldwide overuse of antimicrobial agents has already brought major concerns: the spread of AMR in microorganisms through the global ecosystem. Microbiota of animals, fish, and plants, which have been treated with antimicrobial agents, can serve as a reservoir of resistance genes where commensal microorganisms may confer AMR to pathogenic microorganisms by horizontal transfer events (215). Use of low-dose antibiotics as antimicrobial growth promoters (AGPs) in livestock farming is still a common practice in many countries, which poses a great risk to accelerate emergence and spread of antibiotic-resistant bacteria (216).

It has been shown that antimicrobial agents can alter intestinal microbiota of animals in location-specific ways, (i) structural and functional disruption of foregut microbiota, and (ii) increase of AMR in hindgut microbiota (217). The major risk of disruption of foregut microbiota in piglet is characterized by the increased number of *Streptococcus suis* and Enterobacteriaceae, which are known to cause infectious diseases like pneumonia and post-weaning diarrhea (218). Such effects by the early-life exposure to antimicrobial agents can be retained throughout the life of animals (70, 219). Reduced diversity of intestinal microbiota by antimicrobial treatments, which can increase the host susceptibility to pathogens, has also been documented in fish (220).

The risk of AMR in agriculture is not restricted to livestock farms. Livestock manure is frequently used for composting and eventually amended to agricultural soils as fertilizers and the high frequency of AMR in manure can be transferred to the microbial community in the soil, which can also affect the plant microbiota (221, 222). Aquaculture ponds are considered to be a significant reservoir of AMR (223), especially in countries where livestock manure is used for feeding fish in farming ponds (224). Once entering to the agricultural food chain, AMR is transmitted and exchanged between microbiota associated with animals, fish, and plants and spread over agricultural food products, which can be eventually introduced to human microbiota (225–227).

## Future tasks for defining “optimal microbiota” of animals, fish, and plants

The cross-sectional view of the microbiota of animals, fish, and plants reviewed here may provide an idea of what aspects should be particularly considered in the future investigation for elucidating structure and functions of “optimal microbiota” and applying the knowledge for improving host nutrition and immunology to maximize productivity and sustainability in agriculture.

### Quantitative understanding of microbiota

Many past studies employ 16S rRNA gene sequencing approach to study microbiota of animals, fish, and plants. While the composition of microbiota, which can be estimated by the number of sequencing reads, allows understanding of the diversity and distribution of specific microbial groups, the density of microorganisms is often overlooked. Microbial density is especially important when studying a specific host region where microbial activities play crucial roles or the host immunological factors respond to a certain density threshold. The microbiota of animal rumen, hindguts or rhizosphere with the extremely high density of microorganisms [10^11^−10^12^ cells per mL or gram; (62, 228)] and animal foreguts or the plant phyllosphere with a smaller number of microbial cells in several orders of magnitude (10^4^−10^7^ cells) should be considered as a separate ecosystem themselves, i.e., habitats with different types and levels of microbial structure and functions, which could be differently recognized by the host immunity (162). Therefore, using fecal samples for studying gut microbiota should be done with a special caution, since the foregut microbiota are highly underrepresented in the feces and its compositional and functional changes can be completely masked by the hindgut microbiota (229).

While no direct counts of the number of microbial metabolites produced are available for any host organism, as their composition constantly changes depending on environmental, host and microbial factors, attempts have been made to use sequencing information to estimate the number of compounds that may be produced from human microbiota. Donia et al. have identified 14,000 predicted small-molecule biosynthetic gene clusters (BGCs) by shotgun sequencing human gut metagenome where they have shown that 3,118 BCGs have been found in the healthy human microbiota, among which 599 clusters can be affiliated with typical human gut microbiota while 1,061 clusters with the typical oral cavity (90). They reported that gene cluster classes in the human microbiota differed from those in non-human microbiota, which suggests that species-specific analyses of BCGs will also be useful for agricultural organisms. To make the best use of such useful approach, it is worth summarizing what kind of microbial metabolites can occur and how they affect physiology and growth properties of the host animals, fish, and plants.

### Cultivation of the uncultured majority

The limitation of our current knowledge on microbiota of animals, fish, and plants can be largely attributed to the predominance of uncultured microorganisms in each microbiota. For example, a study by Stanley et al. has been able to identify several bacterial phylotypes in chicken caeca, which are negatively correlated with growth performance of the host chicken, but all of these phylotypes have been affiliated with unknown and uncultured bacterial groups of the phylum Firmicutes (35). In cattle, 44.6% of all microbial sequences obtained from gastrointestinal tracts have failed to be identified at the genus level (3). Similarly, eggs at the fertilization stage of grass carp have been reported to be colonized by a large proportion (>50%) of uncultured bacteria (230). In maize rhizosphere, important functional genes for microbial nitrogen metabolism such as nitrogen-fixation and denitrification have been mainly affiliated with uncultured bacteria (231).

These findings underscore the importance of cultivation of the uncultured members of microbiota colonizing animals, fish, and plants. The difficulties of conventional cultivation techniques are now able to be addressed by modern technologies featured by single-cell (meta)genomics (232) and culturomics (233), which combine the analytical methods such as the index fluorescence-activated cell sorting (FACS) or the matrix-assisted laser desorption/ionization–time of flight (MALDI–TOF) with multiple culture conditions and high-throughput 16S rRNA gene sequencing (233, 234). Isotope probing (SIP) (235) has also been used as a powerful method to identify uncultured microorganisms with specific activities such as host-protein utilization in animal gut microbiota (236) or the pesticide degradation in the rhizosphere microbiota (237). Individual profiles of microbial metabolites (Table 1) are also an important aspect to understand the role of uncultured microorganisms, which can be extensively assessed by recently advancing metabolomic approach integrated with genomic and proteomic datasets (238).

### Systematic investigation of microbial functions

Many well-described Proteobacteria species, which are widely distributed in healthy animals, fish and plants, also behave as opportunistic pathogens (239, 240). *Campylobacter* spp. and *Salmonella* spp., two major food-borne pathobionts have been found to be stable colonizers of livestock animals and human, which are usually unharmful but occasionally cause diseases of the hosts (241). Plant-associated bacteria of the class Gammaproteobacteria such as Pseudomonadaceae, Erwiniaceae, Xanthomonadaceae show species- and strain-level differences in their traits as pathogens, antagonists of the pathogens, or PGPM (240). Most microorganisms consisting the microbiota of animals, fish, and plants seem to be opportunistic symbionts, which colonization can result in beneficial and detrimental, or no effects on the host, which might be determined not only by genetic properties of each microorganism but also by various environmental conditions and host factors (242, 243). The question whether the identified bacterial groups are beneficial or detrimental cannot be answered only by 16S rRNA gene-based analyses, and the microbial metabolites (244), host-specific selective marker genes including virulence or symbiotic factors in the microbial genomes (145, 245), and metabolites involved in modulations of the host cell immunity (246) should be systematically investigated for elucidating the roles of microbiota. A study on the fruit fly *Drosophila* has shown that occurrence of a single protein of plant pathogenic bacterium *Erwinia carotovora*, i.e., *evf* factor determines the successfulness of persistence in the gut of the host (247). Similar unknown mechanisms may present in microorganisms associated with animals, fish, and plants, which are responsible for the host-specific selection of individual microorganisms.

As the majority is still uncultured, microbial physiology is not fully resolved and many important microbial processes in natural ecosystems have still not well-discussed in microbiota research on animals, fish, and plants.

Nitrogen fixation, as well as ammonia oxidation and denitrification (reduction of nitrate, nitrite, and N_2_O), are globally important processes conducted by microorganisms fueling the nitrogen cycle of most ecological systems (248) but have been poorly investigated for animal and fish microbiota. Nitrogen fixation and other inorganic nitrogen conversion have been known to maintain nutritional status of termite gut microbiota, where nitrogen-poor wood polysaccharides (cellulose and hemicellulose) serve as major sources of nutrition (249, 250). Recent findings of a genetic diversity of the nitrogen fixation gene *nifH* in human microbiota indicate that inorganic nitrogen metabolism may play an important role in animal microbiota (251), but it is still unknown which microorganisms are responsible for the processes.

Microbial removal of hydrogen (H_2_) generated in the course of fermentation of fiber-rich carbohydrates is a critical process in every anaerobic system, including gut microbiota of animals and fish (252). H_2_-consuming intestinal microorganisms such as methanogenic archaea, sulfate-reducing bacteria, and reductive acetogens are therefore as important as primary fermenters such as Bacteroidetes for maintaining the redox balance and conserving energy (253), and are crucial for the stable SCFA production in the ecosystem (254, 255). In contrast to the well-studied rumen microbiota, little is known about H_2_-consuming microorganisms for monogastric animal guts, but a study by Rey et al. have shown that genes encoding Wood-Ljundahl pathway, which are key components for reductive acetogenesis, have been shown to be highly represented among expressed RNAs in human gut microbiota than marker genes for methanogenesis or sulfate reduction (256).

### Clarification of optimization purposes

Recently, Lloyd-Price et al. has suggested “healthy human microbiome” can be defined in terms of microbial composition, function, dynamics, and ecology (6). Although this definition can be applied for defining “optimal microbiota” of animals, fish, and plants in agriculture, the dataset from each target organism may be highly limited compared with that of human gut microbiome (257) and additional criteria should be considered in the context of productivity and sustainability.

In plant science, improving growth speeds, conferring resistance against environmental stresses, or improving nutritional values have been successfully accomplished by inoculating PGPM consisting of specific bacterial groups or amending materials promoting the growth of PGPM (258). In contrast, the impact of inoculation of putatively beneficial microorganisms i.e., probiotics to animals and fish seems to be less pronounced (259). Striking similarities between gut microbiota in antibiotic-treated pigs, which gain weight and have high-feed efficiency, and gut microbiota linked to human obesity (218, 260) indicate that the “optimal microbiota” are not necessarily identical to the “healthy microbiota” in agricultural contexts. Therefore, clarifying purposes of the microbiota optimization, i.e., prevention of specific diseases or addition of nutritional values in the products, is prerequisite for the employment of microbiota manipulation techniques, which have been reviewed by Brugman et al., in this issue (11). As discussed above, the worldwide threat of AMR should be combated by reducing the amount of unnecessary use of antimicrobial agents in agricultural practice and by manipulation of microbiota, which can minimize the risk of diseases and optimize the growth performance of target organisms. Host–microbe interaction in individual agricultural organisms should be studied with close reference to the current knowledge available from laboratory models and humans, through which new ideas for modulating microbiota as alternative strategies to antibiotic use can be shared and discussed interdisciplinarily.

## Author contributions

WI-O and CP conceived the idea of the review, WI-O wrote the manuscript, and all authors discussed the contents and contributed to the writing of the manuscript.

### Conflict of interest statement

The authors declare that the research was conducted in the absence of any commercial or financial relationships that could be construed as a potential conflict of interest.
