# Corrigendum: How Can We Define “Optimal Microbiota?”: A Comparative Review of Structure and Functions of Microbiota of Animals, Fish, and Plants in Agriculture

**DOI:** 10.3389/fnut.2018.00113

**Published:** 2018-11-28

**Authors:** Wakako Ikeda-Ohtsubo, Sylvia Brugman, Craig H. Warden, Johanna M. J. Rebel, Gert Folkerts, Corné M. J. Pieterse

**Affiliations:** ^1^Laboratory of Animal Products Chemistry, Graduate School of Agricultural Science, Tohoku University, Sendai, Japan; ^2^Cell Biology and Immunology Group Wageningen University and Research, Wageningen, Netherlands; ^3^Departments of Pediatrics, Neurobiology Physiology and Behavior, University of California, Davis, Davis, CA, United States; ^4^Wageningen Livestock Research Wageningen University and Research, Wageningen, Netherlands; ^5^Division of Pharmacology, Utrecht Institute for Pharmaceutical Sciences, Faculty of Science Utrecht University, Utrecht, Netherlands; ^6^Plant–Microbe Interactions, Department of Biology, Science4Life Utrecht University, Utrecht, Netherlands

**Keywords:** microbiota, agriculture, animal husbandry, aquaculture, rhizosphere, phyllosphere, agricultural immunology

In the original article, we regret that the following Funding statement was missing:

This work was financially supported by the Japan Society for the Promotion of Science (JSPS) through JSPS Core-to-Core Program (Advanced Research Networks) entitled Establishment of international agricultural immunology research-core for a quantum improvement in food safety.

The authors apologize for this error and state that this does not change the scientific conclusions of the article in any way. The original article has been updated.

## Conflict of interest statement

The authors declare that the research was conducted in the absence of any commercial or financial relationships that could be construed as a potential conflict of interest.

